# Supplementary material for: Periodic Revisions of the International Choices Criteria: Process and Results
Source: Nutrients. 2020 Sep 11;12(9):2774. doi: 10.3390/nu12092774 (PMC7551836; doi:10.3390/nu12092774)
Supplement: Supplementary file 1 [file nutrients-12-02774-s001.zip › 05 Sup Table 4 - data in GI2.docx]

| Product group | South Africa | Australia | India | Hong Kong | USA | UK | TOTAL | TOTAL minus excluded products* |
| --- | --- | --- | --- | --- | --- | --- | --- | --- |
| Water (plain), tea, coffee | 7 | 35 | 9 | 15 | 131 | 12 | **209** | **209** |
| Bread | 445 | 1,746 | 171 | 111 | 8,654 | 3,254 | **14,381** | **14,236** |
| Dark sauces | 39 | 141 | 30 | 33 | 169 | 83 | **495** | **495** |
| Processed beans and legumes | 79 | 192 | 48 | 11 | 2,570 | 437 | **3,337** | **3,328** |
| Processed meat, poultry and meat/poultry products | 520 | 1,564 | 78 | 45 | 9,965 | 6,196 | **18,368** | **18,304** |
| Unprocessed meat and poultry | 33 | 201 | 2 | 10 | 582 | 844 | **1,672** | **1,670** |
| Plain rice | 51 | 160 | 63 | 98 | 809 | 261 | **1,442** | **1,395** |
| Flavored rice | 14 | 79 | 1 | 2 | 695 | 234 | **1,025** | **1,024** |
| Plain noodles | 17 | 114 | 15 | 18 | 103 | 81 | **348** | **340** |
| Flavored noodles | 68 | 209 | 119 | 14 | 282 | 287 | **979** | **901** |
| TOTAL | **1,273** | **4,441** | **536** | **357** | **23,960** | **11,689** | **42,256** | **41,902** |

**Supplementary Table 4: Number of products in GI2 database per product group and country**

* Products were excluded if data on one or more of the critical nutrients was lacking, except when nutrient data were lacking for the complete dataset.
