# Supplementary material for: Periodic Revisions of the International Choices Criteria: Process and Results
Source: Nutrients. 2020 Sep 11;12(9):2774. doi: 10.3390/nu12092774 (PMC7551836; doi:10.3390/nu12092774)
Supplement: Supplementary file 1 [file nutrients-12-02774-s001.zip › 04 Sup Table 3 - data in GI1.docx]

| Product group | China | India | Hong Kong | USA | South Africa | UK | New Zealand | Australia | TOTAL | TOTAL minus excluded products* |
| --- | --- | --- | --- | --- | --- | --- | --- | --- | --- | --- |
| Beverages | 197 | 58 | 87 | 771 | 120 | 239 | 227 | 210 | **1,909** | 1,904 |
| Bread | 40 | - | 12 | 411 | 2 | 19 | 37 | 42 | **563** | 502 |
| Bread toppings incl. hummus-type products | 1 | 16 | 5 | 60 | 3 | 20 | 70 | 118 | **293** | 251 |
| Breakfast cereal products | 29 | 24 | 64 | 319 | 21 | 169 | 57 | 62 | **745** | 711 |
| Cheese products | - | - | 33 | 490 | 34 | 79 | 13 | 90 | **739** | 721 |
| Fresh or fresh frozen fruits, vegetables and legumes | - | - | - | 11 | - | - | 43 | 19 | **73** | 73 |
| Fruit juices | 65 | 35 | 27 | 533 | 25 | 179 | 174 | 104 | **1,142** | 1,096 |
| Grains and cereal products – non-wheat | - | - | - | 3 | - | 9 | 2 | 5 | **19** | 19 |
| Grains and cereal products – wheat-based | - | - | - | 2 | - | 4 | 9 | 12 | **27** | 27 |
| Meal sauces | 10 | 7 | 37 | 225 | 113 | 355 | 323 | 293 | **1,363** | 1,314 |
| Meals | 70 | 6 | 14 | 937 | 1 | 68 | 52 | 89 | **1,237** | 1,186 |
| Milk products | 41 | 8 | 76 | 885 | 116 | 307 | 69 | 386 | **1,888** | 1,818 |
| Nuts and seeds | - | 1 | - | 214 | 1 | 5 | 1 | 11 | **233** | 231 |
| Oils, fats and fat-containing spreads | 3 | 9 | 19 | 36 | 17 | 42 | - | 15 | **141** | 135 |
| Other sauces (emulsions) | - | - | 25 | 220 | 19 | 90 | 73 | 89 | **516** | 507 |
| Other sauces (water-based) | 19 | 19 | 57 | 78 | 13 | 88 | 65 | 83 | **422** | 293 |
| Potatoes (processed), pasta and noodles | 71 | 32 | 7 | 174 | 22 | 77 | 45 | 37 | **465** | 360 |
| Processed and dried fruits and vegetables | 14 | - | 2 | 230 | - | 15 | 147 | 76 | **484** | 467 |
| Processed beans and legumes | - | 1 | 2 | 46 | - | 26 | 46 | 28 | **149** | 145 |
| Processed fish or fish products | - | - | - | 5 | - | - | - | - | **5** | 5 |
| Processed meat, meat products and meat substitutes | 3 | - | - | 392 | 18 | 6 | - | 3 | **422** | 415 |
| Rice | - | - | - | 69 | - | 68 | 26 | 21 | **184** | 121 |
| Snacks | 595 | 203 | 316 | 3,742 | 249 | 1,503 | 735 | 1,257 | **8,600** | 7,212 |
| Soups | 19 | 36 | 56 | 379 | 127 | 173 | 22 | 161 | **973** | 635 |
| Water (plain), tea, coffee | 4 | 65 | 7 | 23 | 2 | 2 | 3 | 5 | **111** | 61 |
| TOTAL | **1,181** | **520** | **846** | **10,255** | **903** | **3,543** | **2,239** | **3,216** | **22,703** | **20,209** |

**Supplementary Table 3: Number of products in GI1 database per product group and country**

* Products were excluded if data on one or more of the critical nutrients was lacking, except when nutrient data were lacking for the complete dataset.
