# Supplementary material for: Periodic Revisions of the International Choices Criteria: Process and Results
Source: Nutrients. 2020 Sep 11;12(9):2774. doi: 10.3390/nu12092774 (PMC7551836; doi:10.3390/nu12092774)
Supplement: Supplementary file 1 [file nutrients-12-02774-s001.zip › Supp table 5 Equivalence criteria.docx]

**Supplementary table 5: Equivalence criteria**

*Source: International Choices criteria - A global standard for healthier food (Version 2019-2); Choices International Foundation. Available at www.choicesprogramme.org*

Equivalence criteria are the minimum levels of certain micronutrients in a certain basic product group. They have been defined for situations in which uncertainty arises as to which product group a certain food belongs to. All product groups belonging to the basic foods are supposed to provide a substantial amount of two or more of the vitamins or minerals listed below. The equivalence criteria are always applied for replacements of main products. These are mainly products with the intended use as the product group but does not match the definition of the product group. For other product groups, these equivalence values merely indicate the contents of a product.

An equivalent food product must contain a minimum quantity (see table below) of two of the following nutrients:

- for fruit & vegetables: vitamin A, folic acid, vitamin C and dietary fiber
- for bread and grains: vitamin B1, vitamin B6, folic acid, iron and dietary fiber
- for milk (products): vitamin B2, vitamin B12, folic acid and calcium
- for meat, poultry, fish: vitamin A, vitamin D, vitamin B1, vitamin B12 and iron
- for fats, oils and fat containing spreads: vitamin A, vitamin E, vitamin D
- for fruit & vegetables: vitamin A, folic acid, vitamin C and dietary fiber
- for bread and grains: vitamin B1, vitamin B6, folic acid, iron and dietary fiber
- for milk (products): vitamin B2, vitamin B12, folic acid and calcium
- for meat, poultry, fish: vitamin A, vitamin D, vitamin B1, vitamin B12 and iron
- for fats, oils and fat containing spreads: vitamin A, vitamin E, vitamin D

From at least two of the abovementioned nutrients per product group, a product must contain at least the following amount per 100 grams:

| Nutrient | Value per 100g |
| --- | --- |
| Vitamin A (retinol equivalents) | 70 µg |
| Vitamin E | 1.5 mg |
| Vitamin D | 0.5 µg |
| Vitamin B1 | 0.11 mg |
| Vitamin B2 | 0.11 mg |
| Vitamin B6 | 0.13 mg |
| Vitamin B12 | 0.24 µg |
| Folic acid | 40 µg |
| Vitamin C | 7.5 mg |
| Calcium | 100 mg |
| Iron | 0.8 mg |
| Dietary fiber | 2.5 g |
