# Supplementary material for: Periodic Revisions of the International Choices Criteria: Process and Results
Source: Nutrients. 2020 Sep 11;12(9):2774. doi: 10.3390/nu12092774 (PMC7551836; doi:10.3390/nu12092774)
Supplement: Supplementary file 1 [file nutrients-12-02774-s001.zip › 03 Sup table 2 dietary recommendations.docx]

| Energy | | |
| --- | --- | --- |
| Organization/Country | **Former guideline** (before 2013) | **Current guideline** (since 2013) |
| EFSA^1^ | There was no scientific report on energy intakes | 1800 to 2600 kcal per day depending on age and sex |
| Public Health England^2^ | No specific recommendation on energy intake was set in the previous guideline (more dietary pattern recommendations were involved) | 2000 to 2500 kcal per day |
| Dietary Guidelines for Chinese Residents^3^ | N/A | Varies depending on a number of factors, including a person’s age, sex, height, weight, and level of physical activity |

| Sugar | | |
| --- | --- | --- |
| Organization/Country | **Former guideline** (before 2013) | **Current guideline** (since 2013) |
| WHO International^4^ | “Reducing free sugars intake to less than 10% of total daily energy intake was recommended by the WHO Study Group for the first time in 1989 and was further elaborated by a Joint WHO/FAO Expert Consultation in 2002. This new updated WHO guideline calls for further reduction of free sugars intake to less than 5% of total energy intake if possible.” | <10% of total energy intake  WHO suggests a further reduction of the intake of free sugars to below 5% of total energy intake |
| USDA^5^ | “Reduce the intake of calories from solid fats and added sugars.” | <10% of calories per day |
| Public Health England^2^ | “Cut down on sugar” | <30 grams per day |

| SAFA |
| --- |
| No changes in SAFA recommendations |

| Sodium | | |
| --- | --- | --- |
| Organization/Country | **Former guideline** (before 2013) | **Current guideline** (since 2013) |
| USDA^5^ | <2.3 g/day, <1.5 g/day for people >50 | <2.3 g/day |
| American Heart Association^6^ | N/A | <2.3 g/day |
| Dietary Guidelines for Chinese Residents^3^ | N/A | <2.3 g/day |

| Dietary fiber |
| --- |
| No changes in total fiber recommendations |

| TFA | | | |
| --- | --- | --- | --- |
| Organization/Country | **Former guideline** (before 2013) | | **Current guideline** (since 2013) |
| EFSA^7^ | |  | ‘As low as possible’ |
| WHO^8^ | |  | ‘Less than 1% of total energy intake’ and ‘towards the elimination of industrial trans fats in 2023’ |
| USDA ^5^ | |  | Partially Hydrogenated Oils (PHOs) are not Generally Recognized as Safe (GRAS). |
| The American Heart Association^9^ | |  | ‘As low as possible’ |
| Dietary Guidelines for Chinese Residents^3^ | |  | “Daily intake should be less than 2 g” |

| Total fat |
| --- |
| No changes in total fat recommendations |

**Supplementary Table 2: Changed dietary guidelines and nutrition recommendations since 2013**

1. EFSA sets average requirements for energy intake. *European Food Safety Authority* https://www.efsa.europa.eu/en/press/news/130110 (2013).

2. The Eatwell Guide. *GOV.UK* https://www.gov.uk/government/publications/the-eatwell-guide.

3. Wang, S., Lay, S., Yu, H. & Shen, S. Dietary Guidelines for Chinese Residents (2016): comments and comparisons. *J. Zhejiang Univ. Sci. B* **17**, 649–656 (2016).

4. WHO | Sugars intake for adults and children. *WHO* http://www.who.int/nutrition/publications/guidelines/sugars_intake/en/.

5. 2015-2020 Dietary Guidelines | health.gov. https://health.gov/dietaryguidelines/2015/guidelines/.

6. Horn, L. V. *et al.* Recommended Dietary Pattern to Achieve Adherence to the American Heart Association/American College of Cardiology (AHA/ACC) Guidelines: A Scientific Statement From the American Heart Association. *Circulation* (2016).

7. Scientific and technical assistance on trans fatty acids. *EFSA Support. Publ.* **15**, 1433E (2018).

8. REPLACE trans fat. https://www.who.int/nutrition/topics/replace-transfat.

9. Sacks, F. M. *et al.* Dietary Fats and Cardiovascular Disease: A Presidential Advisory From the American Heart Association. *Circulation* **136**, e1–e23 (2017).
