# Supplementary material for: Periodic Revisions of the International Choices Criteria: Process and Results
Source: Nutrients. 2020 Sep 11;12(9):2774. doi: 10.3390/nu12092774 (PMC7551836; doi:10.3390/nu12092774)
Supplement: Supplementary file 1 [file nutrients-12-02774-s001.zip › 06 Sup fig 2 movable graph example.docx]

**Supplementary figure 2:** Example of a movable graph for the percentage compliance for Processed meat and meat products. Critical nutrient for this product group are SAFA and sodium.

**
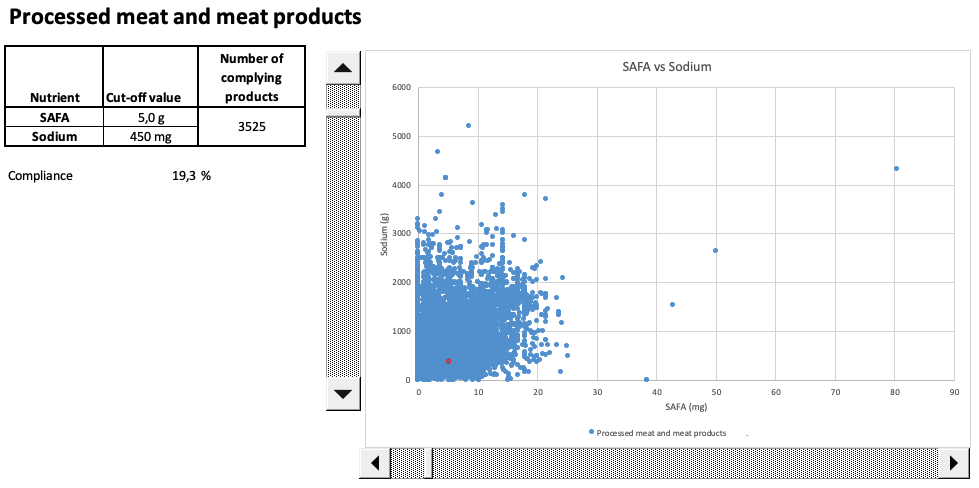
**
