# Supplementary material for: Periodic Revisions of the International Choices Criteria: Process and Results
Source: Nutrients. 2020 Sep 11;12(9):2774. doi: 10.3390/nu12092774 (PMC7551836; doi:10.3390/nu12092774)
Supplement: Supplementary file 1 [file nutrients-12-02774-s001.zip › 02 Sup Figure 1 to grams per 100 gram.docx]

**Supplementary Figure 1: Example of a graph used for conversion of unit of expression**

Products from the product group ‘Main courses’ were plotted within a graph by their SAFA content expressed in en% (y-axis) and g/100g (x-axis). A linear relationship was determined to set the criteria in g/100g. The former cut-off value was 13en%, which corresponds to 2g/100g.

**Main course: en% SAFA vs g/100g**

**
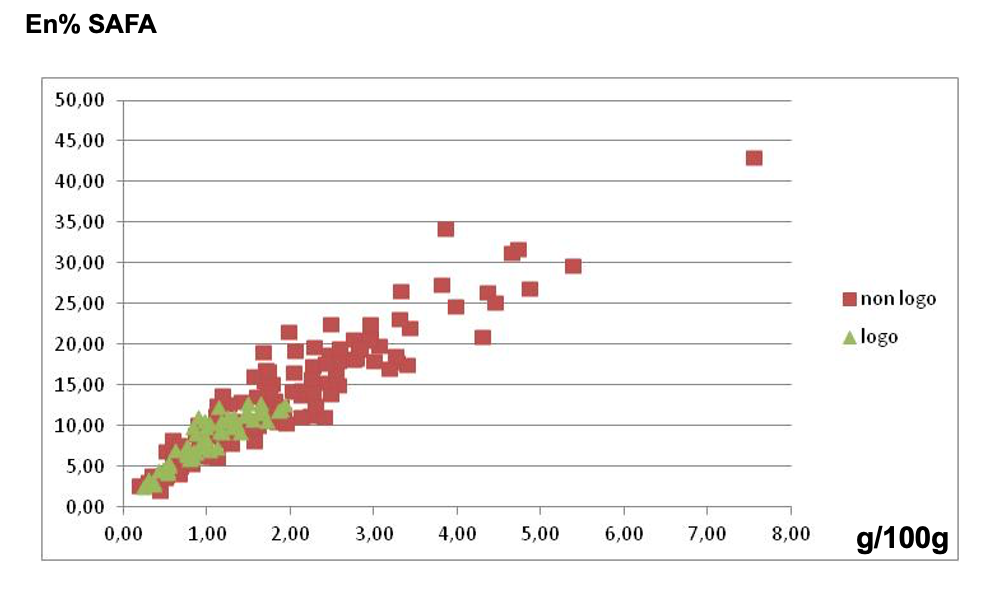
**
